# Dabigatran etexilate for thromboprophylaxis in over 5000 hip or knee replacement patients in a real-world clinical setting

**DOI:** 10.1186/s12959-016-0082-4

**Published:** 2016-04-01

**Authors:** Nadia Rosencher, Charles M. Samama, Martin Feuring, Martina Brueckmann, Eva Kleine, Andreas Clemens, Simon Frostick

**Affiliations:** Department of Anaesthesia and Intensive Care Medicine, Cochin University Hospital, Assistance-Publique – Hôpitaux de Paris; Université Paris Descartes, Paris, France; Faculty of Medicine Mannheim, University of Heidelberg, Mannheim, Germany; Boehringer Ingelheim Pharma GmbH & Co. KG, Ingelheim, Germany; Royal Liverpool University Hospital, Liverpool, L69 3GA UK

**Keywords:** Dabigatran etexilate, Observational study, Thromboprophylaxis, Total hip replacement, Total knee replacement

## Abstract

**Background:**

Thromboprophylaxis is recommended for patients undergoing total hip or total knee replacement (THR, TKR). An international, open-label, prospective, observational, single-arm study in a routine clinical setting was performed to assess the safety and efficacy of dabigatran etexilate 220 mg once daily in patients undergoing THR or TKR, and in subgroups of patients with potentially increased risk of bleeding or venous thromboembolism (VTE).

**Materials and methods:**

Patients were ≥18 years and required to be eligible to receive dabigatran 220 mg once daily (first dose 110 mg 1–4 h after THR/TKR surgery) according to the European Summary of Product Characteristics. The primary safety and efficacy outcomes were incidence of major bleeding events (MBEs), and the composite incidence of symptomatic VTE events and all-cause mortality, respectively.

**Results:**

In total, 5292 patients (median age 64 years) were enrolled and received dabigatran (2734 THR and 2558 TKR). Median drug exposure was 31 days (THR 34 days; TKR 27 days). Overall incidence of MBEs was 0.72 % (95 % confidence interval [CI] 0.51, 0.98), and this rate was comparable between types of surgery and was not significantly affected by protocol-defined risk factors. The overall incidence of symptomatic VTE and all-cause mortality was 1.04 % (95 % CI 0.78, 1.35); the only significant risk factor was history of VTE events (odds ratio 5.59; 95 % CI 2.53, 11.08). A post-hoc analysis showed that the incidence of MBEs in this observational study was similar to or lower than those reported in previous phase 3 trials.

**Conclusions:**

Results from this observational study of dabigatran etexilate administered to patients undergoing THR or TKR surgery are reassuring and supportive of those obtained in dabigatran phase 3 trials.

**Trial registration:**

ClinicalTrials.gov identifier: NCT00846807.

**Electronic supplementary material:**

The online version of this article (doi:10.1186/s12959-016-0082-4) contains supplementary material, which is available to authorized users.

## Background

Patients undergoing total hip or total knee replacement (THR, TKR) are at risk of developing venous thromboembolic events and therefore, thromboprophylaxis is recommended [1]. Compared with low-molecular-weight heparin (LMWH) administered subcutaneously, oral anticoagulants such as dabigatran etexilate (hereafter described as dabigatran) offer important practical advantages. Dabigatran, a low-molecular-weight, reversible thrombin inhibitor, is recommended for primary prevention of venous thromboembolism (VTE) in THR and TKR [1] and has been approved for this indication in more than 100 countries.

Dabigatran is 80 % renally excreted and its terminal half-life is approximately 11–17 h [2, 3]. The European Medicines Agency recommends dabigatran at a dose of 220 mg once daily for the primary prevention of VTE in patients undergoing orthopaedic surgery [3]. For patients with moderate renal impairment, and for those aged ≥75 years or receiving concomitant amiodarone or quinidine, a reduced dose of 150 mg once daily is recommended [3]. Both doses of dabigatran should be initiated 1–4 h after surgery with a half dose to mitigate the bleeding risk in the vulnerable post-operative phase.

The efficacy and safety of dabigatran in the primary prevention of VTE after elective THR or TKR was demonstrated in the phase 3 studies, RE-MODEL [4], RE-MOBILIZE [5], RE-NOVATE [6] and RE-NOVATE II [7]. More than 10,000 patients were randomised in these trials and approximately 6400 received dabigatran. The pooled analysis of the RE-MODEL, RE-MOBILIZE and RE-NOVATE trials showed that dabigatran had similar efficacy to enoxaparin in the prevention of major VTE and VTE-related mortality after knee or hip replacement [8]. The results of the RE-NOVATE II trial were consistent with these findings [7]. No statistically significant differences in the incidence of major bleeding events (MBEs) between treatment groups were found, and no safety concerns regarding elevations in liver function tests or acute coronary syndrome (ACS) events were identified [4, 6–9].

This report provides the results from a large, real-world, observational study of dabigatran in patients undergoing THR or TKR in a routine clinical setting. The purpose of this study was to assess the safety and efficacy of dabigatran 220 mg once daily in all patients who received the drug, as well as in protocol-defined subgroups of patients who had a potentially increased risk of bleeding and/or VTE (i.e. patients with special comorbidities or comedication). This is the first study to provide an insight into the outcomes following use of dabigatran for the prevention of VTE in a real-world orthopaedic setting.

## Methods

### Study design and setting

This was an international, open-label, prospective, observational, single-arm study of patients undergoing elective THR or TKR surgery (clinical trial.gov identifier: NCT00846807). Patients were recruited from 110 sites in nine countries in the European Union. The study was approved by the local institutional ethics committees on human research in the participating centres (Additional file 1: Independent Ethics Committees/Institutional Review Board). All participants provided informed consent.

### Participants and visits

Patients were to be ≥18 years old, undergoing elective THR or TKR and eligible for dabigatran (PRADAXA®, Boehringer Ingelheim, Ingelheim, Germany) 220 mg once daily (first dose 110 mg 1–4 h after surgery) according to the European Summary of Product Characteristics [3] (further details in Additional file 1: Supplementary Methods). Risk factors for VTE events were recorded and patients were assigned to protocol-defined subgroups of interest. These were as follows: active smoking (including patients who interrupted smoking in the peri-operative period [±10 days]); history of coronary artery disease (CAD) or chronic heart failure (CHF; New York Heart Association classification II–IV) ≤6 months before surgery; patients with a history of VTE who were not on any anticoagulant treatment at the time of inclusion in the study; concomitant acetylsalicylic acid (ASA) medication; and patients with chronic use of non-steroidal anti-inflammatory drugs (NSAIDs).

The three assessment visits were a baseline visit ≤7 days before surgery; a second visit, either at discharge from hospital or 24–48 h after the last dose of dabigatran (whichever was the earliest); and a follow-up visit if hospital discharge occurred before the end of treatment (24–48 h after the last dose of dabigatran treatment, e.g. by telephone). All patients provided written informed consent.

### Outcomes

The primary safety endpoint was the incidence of MBEs [10]. These were defined as: clinically overt bleeding associated with a ≥20 g/L fall in haemoglobin in excess of that expected or leading to transfusion of ≥2 units of packed cells/whole blood in excess of that expected; fatal, retroperitoneal, intracranial, intraocular or intraspinal bleeding; or bleeding warranting treatment cessation or leading to re-operation and including wound site (consistent with the Control of Anticoagulation Subcommittee of the International Society on Thrombosis and Haemostasis definition) [10]. Bleeding events that did not meet the criteria for MBE were recorded as any bleeding event.

The primary efficacy variable was the composite of symptomatic VTE (sVTE) (defined as the symptomatic proximal and distal deep vein thrombosis [DVT] and symptomatic nonfatal pulmonary embolism [PE], based on the investigator’s clinical judgement) and all-cause mortality. All VTEs were confirmed via ultrasound, venography or Doppler echocardiography. Nonfatal PEs were all confirmed via pulmonary angiography or ventilation/perfusion (V/Q) scintigraphy.

### Statistical methods

#### Study size

The determination of sample size was based on the anticipated incidence of MBEs and sVTE, which were estimated to be in the range of 0.5 to 2.0 % of the trial population. Based on a sample of 5000 patients, the upper two-sided 95 % confidence limit for the rate estimate would be between 0.74 and 2.43 %. In addition, it was expected that a sample size of 5000 (2500 in each surgery group) would provide at least 400 patients within each of the protocol-defined subgroups. It was estimated that this number of patients would provide 80 % power to detect a rate difference for sVTE and MBE in subgroups of patients with and without protocol-defined risk factors.

#### Analysis

Data were summarised for the entire population by type of surgery and by protocol-defined subgroups. All analyses were descriptive, including *p* values and confidence intervals (CIs) (Clopper-Pearson [11]) and were used for exploratory purposes only. Analyses of study endpoints and adverse events (AEs) focused on the treatment period (i.e. from first dose of dabigatran to 24 h after last dose). Stratified analysis by surgery type was used to assess the confounding of risk factors with surgery type. The association between the presence of protocol-defined risk factors for increased bleeding or VTE and the occurrence of outcome events was assessed using univariable logistic regression analyses, and robustness of the results was checked by running multivariable analyses adjusting for potential confounders (e.g. age). Corresponding CIs and *p* values reported were based on likelihood ratio statistics.

## Results

### Patient disposition

**Fig. 1:**
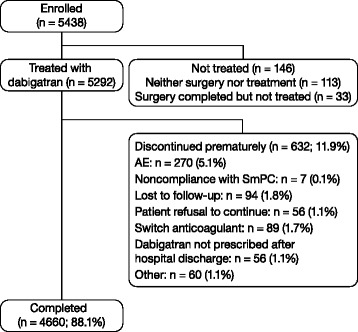
Patient disposition flow diagram. Treated set included 64 noneligible patients. Eligibility to participate and reasons for discontinuation were noted by investigators by completion of tick boxes. *SmPC* Summary of Product Characteristics

This study was conducted from March 2009 to July 2011. In total, 5438 patients were enrolled and 5292 patients were treated (Fig. 1); 88.1 % of treated patients completed dabigatran treatment and the rate of completion was similar for both THR and TKR. The most frequent reason for premature discontinuation was an AE (5.1 %; Fig. 1); the most common AEs leading to discontinuation were events other than bleeding events (3.8 %).

### Baseline characteristics and presence of protocol-defined risk factors

The 5292 treated patients (2734 THR; 2558 TKR) were included in all analyses. Baseline characteristics are shown in Table 1; median drug exposure was 31 days (THR 34 days; TKR 27 days). Of the 5292 patients, 38.2 % had ≥1 protocol-defined risk factor for increased bleeding and/or VTE.

**Table 1:** Baseline patient characteristics – treated set

	THR	TKR	*P* value^a^	Total
Treated, *n*	2734	2558		5292
Male, *n* (%)	1293 (47.3)	938 (36.7)	<0.0001	2231 (42.2)
Age (years), mean (SD)	60.8 (10.22)	64.6 (7.50)	<0.0001	62.6 (9.20)
Age by category			<0.0001	
<65 years, *n* (%)	1586 (58.0)	1117 (43.7)		2703 (51.1)
≥65 years, *n* (%)	1148 (42.0)	1441 (56.3)		2589 (48.9)
BMI (kg/m^2^), mean (SD)	27.8 (4.74)	30.5 (5.25)	<0.0001	29.1 (5.16)
CrCl [mL/min] at visit 1, mean (SD)	109.9 (115.73)	106.1 (70.71)	0.1425	108.1 (96.57)
Medical history				
Any, *n* (%)	1357 (49.6)	1639 (64.1)	<0.0001	2996 (56.6)
Hypertension, *n* (%)	1153 (42.2)	1448 (56.6)	<0.0001	2601 (49.1)
Diabetes mellitus, *n* (%)	207 (7.6)	324 (12.7)	<0.0001	531 (10.0)
Cancer, *n* (%)	179 (6.5)	197 (7.7)	0.1024	376 (7.1)
Liver disease, *n* (%)	59 (2.2)	64 (2.5)	0.4066	123 (2.3)
Renal disease, *n* (%)	44 (1.6)	73 (2.9)	0.0021	117 (2.2)
TIA/stroke, *n* (%)	44 (1.6)	54 (2.1)	0.1761	98 (1.9)
MI, *n* (%)	33 (1.2)	58 (2.3)	0.0030	91 (1.7)
GI bleeding, *n* (%)	23 (0.8)	42 (1.6)	0.0082	65 (1.2)
Immobilisation within the last 10 days	6 (0.2)	5 (0.2)	0.8481	11 (0.2)
Risk factor subgroups				
Active smoker, *n* (%)	465 (17.0)	239 (9.3)	<0.0001	704 (13.3)
CAD, *n* (%)	132 (4.8)	187 (7.3)	0.0001	319 (6.0)
CHF, *n* (%)	54 (2.0)	73 (2.9)	0.0369	127 (2.4)
History of VTE, *n* (%)	73 (2.7)	113 (4.4)	0.0006	186 (3.5)
Concomitant ASA, *n* (%)	166 (6.1)	252 (9.9)	<0.0001	418 (7.9)
Chronic use of NSAIDs, *n* (%)	415 (15.2)	394 (15.4)	0.8214	809 (15.3)

^a^In case of continuous variables *t*-test is applied; for categorical variables chi-square test is applied

*ASA* acetylsalicylic acid, *BMI* body mass index, *CAD* coronary artery disease, *CHF* chronic heart failure, *CrCl* creatinine clearance, *GI* gastrointestinal, *MI* myocardial infarction, *NSAIDs* non-steroidal anti-inflammatory drugs, *SD* standard deviation, *THR* total hip replacement, *TIA* transient ischaemic attack, *TKR* total knee replacement, *VTE* venous thromboembolism

Mean creatinine clearance (CrCl) was 108.1 mL/min (Table 1). The large standard deviation was primarily driven by extremely high CrCl values. Only 1.1 % of patients had moderate renal impairment (due to exclusion criteria for the study). On average, patients undergoing TKR were older than those undergoing THR (mean age was 65 vs 61 years) and had slightly higher body mass index (BMI) (Table 1). In the THR group there were fewer patients with any medical history compared with the TKR group (*p* < 0.0001); this was reflected by fewer patients with a history of hypertension, diabetes mellitus, renal disease, myocardial infarction and gastrointestinal bleeding. The most commonly used cardiovascular medications were agents acting on the renin–angiotensin system (20.6 %). In total, 3378 (63.8 %) patients used ≥1 concomitant nondrug therapy (elastic compression stockings [52.9 %], other therapies [12.5 %] or surgical therapies and interventions [2.0 %]). The use of such therapies was similar for the two surgery groups.

The most common of the protocol-defined factors were chronic use of NSAIDs, active smoking, concomitant ASA use and CAD. Concomitant users of ASA were fewer in the THR group than in the TKR group but the number of active smokers was significantly higher (Table 1). In the THR group, there were also significantly fewer patients with a history of CAD, CHF and VTE compared with the TKR group.

### Safety outcomes

**Fig. 2:**
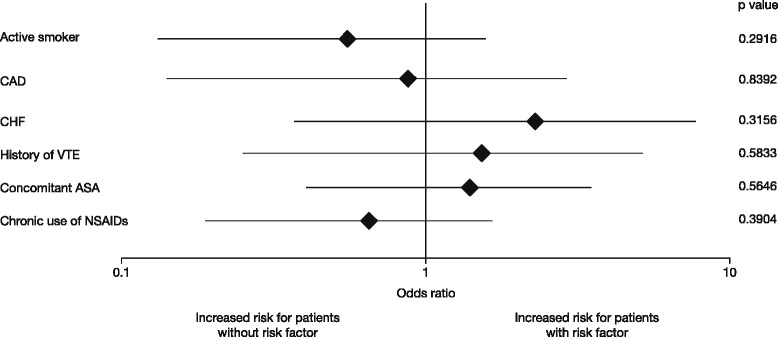
Effect of protocol-defined risk factors on the incidence of MBEs – odds ratios (95 % CI). Odds ratios, based on univariable logistic regression, were calculated for presence versus absence of risk factor; CIs and *p* values are based on likelihood-ratio statistics. *ASA* acetylsalicylic acid, *CAD* coronary artery disease, *CHF* chronic heart failure, *CI* confidence interval, *MBE* major bleeding event, *NSAIDs* non-steroidal anti-inflammatory drugs, *VTE* venous thromboembolism

The overall incidence of MBEs was 0.72 % (95 % CI 0.51, 0.98) (Table 2 and Additional file 1: Supplementary Table 1). Protocol-defined risk factors (Table 2, Fig. 2) and type of surgery (Table 2) had no significant effect on the occurrence of MBEs. When comparing patients with at least one risk factor with patients with none of the risk factors, there was a comparable incidence of MBEs (0.64 % and 0.76 %, respectively). Sensitivity analyses, including several covariates in the logistic regression model, support these results (results not shown).

**Table 2:** Incidence of MBEs overall and in protocol-defined risk factor subgroups – treated set

	THR				TKR				Total			
Risk factor subgroups	*N*	*n*	Incidence %	95 % CI	*N*	*n*	Incidence %	95 % CI	*N*	*n*	Incidence %	95 % CI
All treated	2734	19	0.69	0.42, 1.08	2558	19	0.74	0.45, 1.16	5292	38	0.72	0.51, 0.98
Active smoker												
No	2269	16	0.71	0.40, 1.14	2319	19	0.82	0.49, 1.28	4588	35	0.76	0.53, 1.06
Yes	465	3	0.65	0.13, 1.87	239	0	0	0.00, 1.53	704	3	0.43	0.09, 1.24
CAD												
No	2602	18	0.69	0.41, 1.09	2371	18	0.76	0.45, 1.20	4973	36	0.72	0.51, 1.00
Yes	132	1	0.76	0.02, 4.15	187	1	0.53	0.01, 2.94	319	2	0.63	0.08, 2.25
CHF												
No	2680	19	0.71	0.43, 1.10	2485	17	0.68	0.40, 1.09	5165	36	0.70	0.49, 0.96
Yes	54	0	0	0.00, 6.60	73	2	2.74	0.33, 9.55	127	2	1.57	0.19, 5.57
History of VTE												
No	2661	19	0.71	0.43, 1.11	2445	17	0.70	0.41, 1.11	5106	36	0.71	0.49, 0.97
Yes	73	0	0	0.00, 4.93	113	2	1.77	0.22, 6.25	186	2	1.08	0.13, 3.83
Concomitant ASA												
No	2568	17	0.66	0.39, 1.06	2306	17	0.74	0.43, 1.18	4874	34	0.70	0.48, 0.97
Yes	166	2	1.20	0.15, 4.28	252	2	0.79	0.10, 2.84	418	4	0.96	0.26, 2.43
Chronic use of NSAIDs												
No	2319	17	0.73	0.43, 1.17	2164	17	0.79	0.46, 1.25	4483	34	0.76	0.53, 1. 06
Yes	415	2	0.48	0.06, 1.73	394	2	0.51	0.06, 1.82	809	4	0.49	0.13, 1.26

*N*: number of patients; *n*: number of patients with major bleeding events (MBE)

*ASA* acetylsalicylic acid, *CAD* coronary artery disease, *CHF* chronic heart failure, *CI* confidence interval, *NSAIDs* non-steroidal anti-inflammatory drugs, *THR* total hip replacement, *TKR* total knee replacement, *VTE* venous thromboembolism

**Fig. 3:**
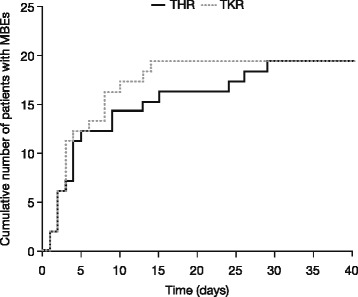
Cumulative incidence of MBEs over time from the start of treatment, treated set. No events occurred after day 29 in THR patients or day 14 in TKR patients. *MBE* major bleeding event, *THR* total hip replacement, *TKR* total knee replacement

In patients who had MBEs (*n* = 38), the onset of the events occurred during the first 7 days of treatment in 63.2 % (12/19) of patients in the THR group and 73.7 % (14/19) in the TKR group. Onset occurred between 8 and 14 days after treatment initiation in 15.8 % (3/19) and 26.3 % (5/19) in the THR and TKR groups, respectively, and >14 days after treatment initiation in 21.1 % (4/19) in the THR group and none of the TKR group. Figure 3 shows the cumulative incidence of MBEs over time.

The overall incidence of any bleeding events was 3.82 % (95 % CI 3.32, 4.37), with a slightly higher incidence for knee surgery (4.61 %) compared with hip surgery (3.07 %) patients. There were no fatal bleeds in the study.

### Efficacy outcomes

**Fig. 4:**
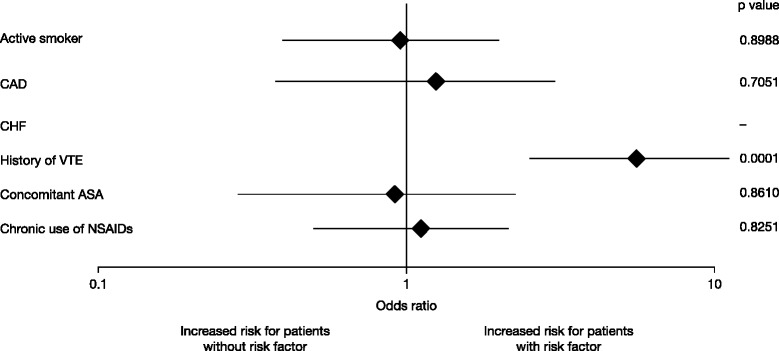
Effect of pre-specified risk factors on the incidence of sVTE and all-cause mortality, treated set. No events occurred in patients with CHF. Odds ratios, based on univariable logistic regression, were calculated for presence versus absence of risk factor; CIs and *p* values are based on likelihood-ratio statistics. *ASA* acetylsalicylic acid, *CAD* coronary artery disease, *CHF* chronic heart failure, *CI* confidence interval, *NSAIDs* non-steroidal anti-inflammatory drugs, *sVTE* symptomatic venous thromboembolism

The overall incidence of symptomatic VTE and all-cause mortality was 1.04 % (95 % CI 0.78, 1.35). With the exception of patients with a history of VTE (a known risk factor for recurrent VTE), there was no relevant difference in the incidence in patients with or without the protocol-defined risk factors (Table 3 and Fig. 4). Patients with a history of VTE had a higher incidence of the primary endpoint (4.84 %) than patients with no history of VTE (0.90 %); odds ratio for the composite endpoint for patients with versus those without a history of VTE was 5.59 (95 % CI 2.53, 11.08; *p* = 0.0001). The latter was driven by events in the TKR patients with a history of VTE (Table 3). Sensitivity analyses, including multiple covariates in the logistic regression model, consistently supported results from the univariate logistic regression.

**Table 3:** Occurrence of primary efficacy endpoint overall and by protocol-defined subgroup – treated set

	THR				TKR				Total			
Risk factor subgroups	*N*	*n*	Incidence %	95 % CI	*N*	n	Incidence %	95 % CI	*N*	*n*	Incidence %	95 % CI
All treated	2734	15	0.55	0.31, 0.90	2558	40	1.56	1.12, 2.12	5292	55	1.04	0.78, 1.35
Active smoker												
No	2269	13	0.57	0.31, 0.98	2319	35	1.51	1.05, 2.09	4588	48	1.05	0.77, 1.38
Yes	465	2	0.43	0.05, 1.54	239	5	2.09	0.68, 4.81	704	7	0.99	0.40, 2.04
CAD												
No	2602	13	0.50	0.27, 0.85	2371	38	1.60	1.14, 2.19	4973	51	1.03	0.76, 1.35
Yes	132	2	1.52	0.18, 5.37	187	2	1.07	0.13, 3.81	319	4	1.25	0.34, 3.18
CHF												
No	2680	15	0.56	0.31, 0.92	2485	40	1.61	1.15, 2.19	5165	55	1.06	0.80, 1.38
Yes	54	0	0	0.00, 6.60	73	0	0	0.00, 4.93	127	0	0	0.00, 2.86
History of VTE												
No	2661	15	0.56	0.32, 0.93	2445	31	1.27	0.86, 1.79	5106	46	0.90	0.66, 1.20
Yes	73	0	0	0.00, 4.93	113	9	7.96	3.71, 14.58	186	9	4.84	2.24, 8.99
Concomitant ASA												
No	2568	12	0.47	0.24, 0.81	2306	39	1.69	1.21, 2.30	4874	51	1.05	0.78, 1.37
Yes	166	3	1.81	0.37, 5.19	252	1	0.40	0.01, 2.19	418	4	0.96	0.26, 2.43
Chronic use of NSAIDs												
No	2319	13	0.56	0.30, 0.96	2164	33	1.52	1.05, 2.13	4483	46	1.03	0.75, 1.37
Yes	415	2	0.48	0.06, 1.73	394	7	1.78	0.72, 3.63	809	9	1.11	0.51, 2.10

*N* number of patients, *n* number of patients with symptomatic VTE or who died of any cause

*ASA* acetylsalicylic acid, *CAD* coronary heart disease, *CHF* chronic heart failure, *CI* confidence interval, *NSAIDs* non-steroidal anti-inflammatory drugs, *THR* total hip replacement, *TKR* total knee replacement, *VTE* venous thromboembolism

The incidence of the composite efficacy endpoint was lower for THR patients (0.55 %) than for patients undergoing knee surgery (1.56 %) (Table 3). The most frequent contributor to the primary efficacy endpoint was symptomatic distal DVT (39 events: 7/15 THR patients and 32/40 TKR patients with an event had a symptomatic distal DVT).

**Fig. 5:**
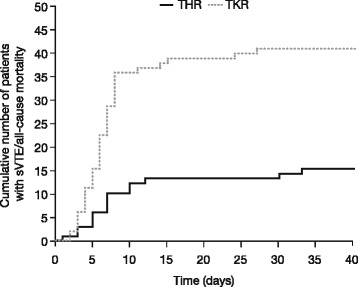
Cumulative incidence of sVTE or all-cause mortality over time from the start of treatment, treated set. No new events occurred after day 33 in THR patients or day 27 in TKR patients. *sVTE* symptomatic venous thromboembolism, *THR* total hip replacement, *TKR* total knee replacement

In patients with sVTE or all-cause mortality (*n* = 55), event onset occurred within the first 7 days of treatment in 66.7 % (10/15) in the THR group and 70 % (28/40) in the TKR group. Event onset occurred between 8 and 14 days after treatment initiation in 20 % (3/15) in the THR group and 22.5 % (9/40) in the TKR group, and >14 days after treatment initiation in 13.3 % (2/15) in the THR group and 7.5 % (3/40) in the TKR group. Figure 5 shows the cumulative incidence of sVTE or all-cause mortality over time.

### Adverse events

Overall, treatment-emergent AEs (including bleeding and efficacy outcome events) were observed in 987 (18.7 %) of the 5292 patients. Nausea (2.6 %), vomiting (1.7 %), pyrexia (1.3 %) and operative haemorrhage (1.3 %) were the only AEs observed in >1 % of patients. Adverse events were considered drug-related by the investigator in 5.5 % of patients; operative haemorrhage (1.2 %) was the only drug-related AE reported in >0.5 % of patients.

Three patients died during the study. One death due to PE was considered by the investigator to be related to the study drug. The other two, reported only as ‘death’, were not considered by the investigator to be drug-related. Serious AEs were reported in 2.6 % of patients; the most frequently reported (in 0.2 % each) were DVT, PE, device dislocation and ineffective drug, as reported by the investigators. AEs resulted in discontinuation of the study drug in 4.9 % of patients; DVT (0.6 %) was the only AE that resulted in discontinuation of >0.5 % of patients. No safety concerns regarding ACS events were identified overall (either during treatment or in the post-study period) (Additional file 1: Supplementary Table 2).

## Discussion

Results from this observational study showed that there is no increased risk of bleeding with dabigatran 220 mg once daily in patients undergoing THR or TKR (irrespective of the presence or absence of protocol-defined risk factors for bleeding) in a real-world clinical setting compared with the phase 3 clinical trials [4, 6, 7]. A history of VTE, a known risk factor for subsequent VTE [12], was the only protocol-defined characteristic associated with an increased primary efficacy event rate (sVTE or death). A higher event rate in patients with this risk factor was observed for those undergoing TKR (7.96 %) but not for those undergoing THR (0 %).

The American College of Chest Physicians (ACCP) guidelines estimated that, from 0 to 35 days, the cumulative post-operative rate of nonfatal sVTE in patients undergoing major orthopaedic surgery is 4.3 % in the absence of prophylaxis and 1.8 % with an LMWH [1]. The incidence of the primary endpoint in this study, which included fatal as well as nonfatal VTE events, was 1.04 % and therefore lower than the ACCP estimates when patients were treated with an LMWH. Although several factors may have influenced the incidence of sVTE events and bleeding events, it is worth noting that characteristics of the patients included in this observational study are broadly aligned with previously published real-world data sets [13–15].

The primary efficacy endpoint is not directly comparable between this observational study and the phase 3 trials. In the observational study the primary endpoint was sVTE and all-cause death during the treatment period and the rate was primarily driven by the occurrence of symptomatic distal DVTs. The primary endpoint in the phase 3 studies, RE-MODEL, RE-NOVATE and RE-NOVATE II, was total VTE and all-cause mortality, and included events observed during routine venography.

To explore the data more fully, we performed a post-hoc analysis to facilitate closer comparison between this observational study and the phase 3 results. To lessen the differences introduced by the study designs, only patients ≤75 years of age, from outside the United States and Canada, treated with dabigatran 220 mg once daily with documented information on medication start date and CrCl >50 mL/min, were included. Based on medical judgement, the patient populations were similar in the observational study and the phase 3 TKR and THR trials of dabigatran, apart from a lower incidence of CAD at baseline and a lower concomitant use of NSAIDs in the observational, real-world setting study. Analysis in these comparable populations (4999 patients from the observational study and 2118 from the phase 3 trials) showed that safety and efficacy of dabigatran in this observational study were as favourable as those obtained in the phase 3 dabigatran clinical trials. There was a similar or lower risk of an MBE in this observational study compared with the phase 3 trials in this post-hoc comparison: 0.62 % (95 % CI 0.36, 1.01) for THR and 0.74 % (95 % CI 0.44, 1.17) for TKR in the observational study compared with 0.65 % (95 % CI 0.24, 1.41) in RE-NOVATE and 0.57 % (95 % CI 0.16, 1.46) in RE-NOVATE II for THR, and 1.20 % (95 % CI 0.44, 2.58) in RE-MODEL for TKR. In the same analysis, the incidence of the composite of sVTE and all-cause mortality in the observational study was 0.50 % (95 % CI 0.27, 0.86) for THR and 1.40 % (95 % CI 0.97, 1.96) for TKR. This was intermediate between the results from RE-NOVATE (1.20 % [95 % CI 0.60, 2.13]) and RE-NOVATE II (0.14 % [95 % CI 0.00, 0.08]) for THR, and higher than the results from RE-MODEL (0.20 % [95 % CI 0.01, 1.10]) for TKR. Differences in data collection methods (especially central adjudication of events vs local investigator assessment), patient populations and definitions of efficacy outcome events in the observational study and the phase 3 trials should be considered when interpreting the results from this post-hoc analysis.

Contrary to the protocol recommendation and to ACCP recommendations, a subset of sites performed routine ultrasound examinations to detect VTE; asymptomatic as well as sVTE events could therefore have been reported. We noticed in a stratified analysis by country that the incidence of sVTE and all-cause mortality was higher in France (2.15 %), where duplex sonography is routine clinical practice in some institutions [16], than in other countries (0–1.37 %). Within France, the incidence was highest for patients undergoing knee surgery (4.38 % vs 0.67 % for hip surgery patients). A further post-hoc analysis showed that the incidence of sVTE and all-cause mortality was lower when patients from sites that routinely performed ultrasound examinations were excluded (*n* = 405); the incidence of sVTE and all-cause mortality decreased to 0.86 % (95 % CI 0.62, 1.16) in the remaining 4887 patients (1.12 % in TKR patients [95 % CI 0.74, 1.63]; and 0.60 % in THR patients [95 % CI 0.34, 1.00]). There was a significant effect of diagnosis using routine ultrasound on the reported incidence of sVTE and all-cause mortality (odds ratio 3.83 for sites using vs not using routine ultrasound [95 % CI 1.96, 6.99; *p* = 0.0002]). This difference was driven by a lower incidence of patients with distal symptomatic DVT. Diagnosis using routine ultrasound may therefore explain the higher event rates in TKR patients compared with the rate obtained in phase 3 clinical trials.

It is unclear whether the low incidence of symptomatic VTE and all-cause mortality in THR patients with a history of VTE (0 % compared with 0.56 % in those without a history of VTE) has a medical explanation. It may be a ‘chance’ finding due to sample size. Only 73 patients with a history of VTE underwent hip surgery, so even if the risk increase (due to history of VTE) were as high as that observed for the knee surgery group, we would only expect two to three patients with an event in the THR patients with history of VTE. The use of duplex sonography of the calf in some study centres may also have contributed to the relatively greater difference in the incidence of the primary efficacy outcome between the TKR patients with and without a history of VTE (7.96 % vs 1.27 %). Asymptomatic VTE could be interpreted as symptomatic VTE in knee surgery because of calf haematoma and oedema, but in hip surgery, there is no calf haematoma and thus no possibility to attribute an asymptomatic DVT to a symptomatic event.

Further potential limitations of this observational study include a lack of control group, diagnosis based only on the investigator’s clinical judgement (which could have resulted in false positive or negative findings) and a possible bias in the selection of patients. The expected sample size ≥400 patients was not reached in all subgroups, therefore, the power to detect significant differences in rates on a descriptive basis was decreased, particularly for the CAD and CHF groups. However, the point estimates did not indicate any relevant increase in risk in these groups. A formal comparison of outcomes between THR and TKR surgery groups was not considered worthwhile because of significant differences in patient characteristics and the expectation of different outcomes based on the body of previously published findings.

It is important to note that the favourable AE profile in this observational study was comparable with that obtained in the dabigatran phase 3 trials. No increased ACS signal was detected with dabigatran compared with enoxaparin during or after treatment in a pooled analysis of pivotal phase 3 dabigatran trials [9]. Results from this observational study confirm the low incidence of ACS during dabigatran treatment in a real-world orthopaedic setting.

## Conclusion

The risk factors that were evaluated in the protocol-defined subgroups had little impact on the incidence of major bleeding in patients undergoing TKR or THR in a routine clinical setting. With the exception of a history of VTE, the protocol-defined risk factors for bleeding did not have an impact on the incidence of sVTE and overall mortality. Results from this real-world observational study are reassuring and supportive of the safety and efficacy results obtained in dabigatran phase 3 clinical trials.

## Additional file

Additional file 1: Supplementary Appendix. (DOCX 30.2 kb)

## Abbreviations

ACCP: American College of Chest Physicians
ACS: acute coronary syndrome
AEs: adverse events
ASA: acetylsalicylic acid
BMI: body mass index
CAD: coronary artery disease
CHF: chronic heart failure
CI: confidence interval
CrCl: creatinine clearance
DVT: deep vein thrombosis
GI: gastrointestinal
LMWH: low-molecular-weight heparin
MBEs: major bleeding events
MI: myocardial infarction
NSAIDs: non-steroidal anti-inflammatory drugs
PE: pulmonary embolism
SmPC: Summary of Product Characteristics
sVTE: symptomatic VTE
THR: total hip replacement
TIA: transient ischaemic attack
TKR: total knee replacement
VTE: venous thromboembolism
