# Supplementary material for: Dabigatran etexilate for thromboprophylaxis in over 5000 hip or knee replacement patients in a real-world clinical setting
Source: Thromb J. 2016 Apr 1;14:8. doi: 10.1186/s12959-016-0082-4 (PMC4818472; doi:10.1186/s12959-016-0082-4)
Supplement: Additional file 1: — Supplementary Appendix. (DOCX 30.2 kb) [file 12959_2016_82_MOESM1_ESM.docx]

**Supplementary Appendix**

**Independent Ethics Committees/Institutional Review Boards**

**Lead IECs/IRBs^[[1]](#footnote-1)^ providing approval of the study protocol in each participating country**

Univ. Prof. Dr. Ernst Singer, Ethikkommission der Medizinischen, Universität Wien – AKH, Borschkegasse, 8b/E06, A-1090 Wien, Austria.

Michèle Guillemot, Ministère de L’Enseignement Supérieur et de la Reserche, 1 rue Descartes – 75231 Paris, Cedex 05, France.

Alex Tűrk, Commission Nationale de L’Informatique et des Libertés, 8 rue Vivienne – CS 30223 75083 Paris, Cedex 02, France.

Dr. T Aleker and Dr. G Hook, Ethikkommission der Landesärztekammer, Baden-Württemberg, Jahnstraße 40, 70597 Stuttgart, Germany.

Ms Maire Hickey-Dwyer, Research Ethics Committee, Mid-Western Regional Hospital, Dooradoyle, Co. Limerick, Ireland.

Prof. Armando Savignano, Comitato Indipendente di Etica, Azienda Ospedaliera “Carlo Poma”, V. le Albertoni 1, 46100 Mantova, Italy.

Dr. Francesco Luigi Meloni, Comitato Etico, Azienda Ospedaliera Universitaria “San Martino”, Largo R. Benzi 10, 16132, Genova, Italy.

Prof. Piero Cioni, Comitato Etico Locale, Azienda Ospedaliero Universitaria Careggi, Largo Palagi 1, 50139 Firenze, Italy.

Prof. Alvaro Carotti, Comitato Etico, ASUR Marche Zona Territoriale 5, Via Gallodoro 68, 60035 Jesi, Italy.

Avv. Gian Domenico Caiazza, Comitato Etico, Azienda Ospedaliera Sant’Andrea, Via di Grottarossa 1035, 00189 Roma, Italy.

Prof.ssa Maria Chiara Cassone, Comitato Etico, ASL TO2, Corso Svizzera 185/bis, 10149 Torino, Italy.

Dr. Silvio Chierichetti, Comitato Etico, Azienda Ospedaliera di Desio e Vimercate, Via Cesare Battisti 23, 20059, Vimercate, Italy.

Prof.ssa Gabriella Serio, Comitato Etico, Azienda Sanitaria Locale Provinciale di Bari, Lungomare Starita 6, 70123 Bari, Italy.

Dr. Francesco Vassallo, Comitato Etico, ASL Brescia, Via Galileo Galilei 20, 25128 Brescia, Italy.

Prof. Lorenzo Amici, Comitato Etico, AUSL Latina, Italy.

Prof. Claudio Banterle, Comitato Etico, Azienda Ospedaliera di Desenzano del Garda, Località Montecroce, 25015 Desenzano del Garda, Italy.

Dr. habil. med. Jarosław Reguła, Bioethic Committee at CMKP, ul. Marymoncka 99/103, 01-813 Warszawa, Poland.

Vincent Valentín Segura, Comité Ético de Investigación Clínica del H. Univ. Dr. Peset, Valencia, Spain.

Eva López Hernández, CEICA Aragón, Spain.

Consuelo María Rodríguez Jiménez, Comité Ético de Investigación Clínica CEIC del Hospital Universitario de Canarias, Spain.

José D. García Labajo, Comité de Ética en Investigación Clínica, Hospital RUBER Internacional, Madrid, Spain.

Antonio Velázquez Martínez, Javier Becares, and Nicolás García González, Spain (no approval required for an observational trial, only notification).

Ricardo Perea Martín, Comite Etico de Investigacion Clinica, HGU Gregorio Marañon, Madrid, Spain.

Mr Lars Dahlstedt, Regionala etikprövningsnämnden i Linköping, c/o Hälsouniversitetets kansli, Sandbäcksgatan 7, 581 83 Linköping, Sweden.

Mr Francis Chan, North West 8 REC – GM East Room 181, Gateway House, Piccadilly South, Manchester M60 7LP, UK.

**Supplementary Methods**

**Exclusion criteria**

According to the label recommendations for 220 mg once daily dabigatran, patients were excluded if they were ≥75 years old, were using amiodarone, had creatinine clearance (Cockcroft-Gault formula) ≤50 mL/min, elevated liver enzymes >2× the upper limit of normal, hepatic impairment or liver disease expected to have any impact on survival, anaesthesia with post-operative indwelling epidural catheters, hypersensitivity to dabigatran, active clinically significant bleeding or an organic lesion at risk of bleeding, a spontaneous or pharmacological impairment of haemostasis, an indication for concomitant treatment with quinidine or chronic anticoagulant therapy.

**Supplementary Table 1** Summary of MBEs by qualifying major bleeding event criteria and by bleeding site – treated set

|  | **THR** | **TKR** | **Total** |
| --- | --- | --- | --- |
| Treated, *n* | 2734 | 2558 | 5292 |
| Number of patients with any MBE, *n* (%) | 19 (0.7) | 19 (0.7) | 38 (0.7) |
| Number of MBEs | 20 | 20 | 40 |
| MBE category^a^ |  |  |  |
| Fatal bleeding | 0 | 0 | 0 |
| ≥20 g/L fall in haemoglobin^b^ | 5 | 8 | 13 |
| Leading to transfusion of ≥2 units | 11 | 4 | 15 |
| Symptomatic bleeding in critical organ^c^ | 1 | 0 | 1 |
| Requiring treatment cessation | 9 | 8 | 17 |
| Leading to re-operation | 4 | 5 | 9 |
| Site of bleeding^d^ |  |  |  |
| Surgical site | 10 | 12 | 22 |
| Intra-articular | 0 | 2 | 2 |
| Gastrointestinal | 9 | 4 | 13 |
| Nasal | 1 | 0 | 1 |
| Other | 0 | 2 | 2 |

*MBEs* major bleeding events, *THR* total hip replacement, *TKR* total knee replacement

^a^Events may be counted in more than one category; ^b^In excess of what the investigator expected; ^c^Symptomatic documented retroperitoneal, intracranial, intraocular or intraspinal bleeding (note: for one patient, the site of bleeding was reported as ‘gastrointestinal’ but the category of event was ‘symptomatic bleeding in critical organ’); ^d^Categories with no entries are not tabulated here

**Supplementary Table 2** Treatment-emergent and post-study ACS events identified by investigator assessment (based on AE data)

|  | ***n*** | **Incidence, %** |
| --- | --- | --- |
| Total patients with ≥1 ACS event during treatment period (total *n* = 5292)  Myocardial infarction  Myocardial ischaemia  Total patients with ≥1 ACS event during post-study period (total *n* = 4421)  Myocardial infarction | 4  3  1  1  1 | 0.1  0.1  0.0  0.0  0.0 |

*ACS* acute coronary syndrome, *AE* adverse event

Percentages are calculated using total number of patients per treatment as the denominator. For dabigatran dose groups, a patient is assigned to the group of his/her first full dose

1. According to French law, only biomedical research needs an approval from an IRB/IEC, called *Comité de protection des personnes* (CPP) in France. Observational studies do not need the approval of a CPP. Two national authorities, the *Comité consultatif sur le traitement de l’information en matière de recherche dans le domaine de la santé* (CCTIRS) and the *Commission nationale de l’informatique et deslibertés* (CNIL), are responsible for authorizing or rejecting entry of data identifying individuals. [↑](#footnote-ref-1)
